# Editorial: Dysregulation of immunity predisposing to severe COVID-19 infection

**DOI:** 10.3389/fimmu.2022.1099089

**Published:** 2022-12-02

**Authors:** Rabih Halwani, Federica Pulvirenti, Saleh Al-Muhsen

**Affiliations:** ^1^ Research Institute of Medical & Health Sciences, University of Sharjah, Sharjah, United Arab Emirates; ^2^ Department of Clinical Sciences, College of Medicine, University of Sharjah, Sharjah, United Arab Emirates; ^3^ Prince Abdullah Ben Khaled Celiac Disease Research Chair, Department of Pediatrics, Faculty of Medicine, King Saud University, Riyadh, Saudi Arabia; ^4^ Reference Centre for Primary Immune Deficiencies, Azienda Ospedaliera Universitaria Policlinico Umberto I, Rome, Italy; ^5^ Immunology Research Laboratory, Department of Pediatrics, College of Medicine, King Saud University, Riyadh, Saudi Arabia

**Keywords:** COVID-19, inborn errors of immunity, SARS-CoV-2, primary immunodeficiency, biomarker

Severe acute respiratory syndrome coronavirus 2 (SARS-CoV-2), the virus causing coronavirus disease 2019 (COVID-19), has already infected over 600 million individuals and claimed at least 6.5 million lives (1). COVID-19 pathogenesis is characterized with pounced inflammation, muted interferon and antiviral immunity, ineffective viral clearance, T cell exhaustion, and delayed adaptive response (2–5).

Global presentation of this infection varied with different individuals, different genders, age groups, and comorbidities, while its severity level ranged from asymptomatic infection to life-threatening disease (4–6). The observed difference in the immune response to SARS-CoV-2 could be due to variation in one of more of host factors including the genetic make-up, immune system age, and immune-metabolic balance (4, 7).

Moreover, Inborn Errors of Immunity (IEI) could increase the host vulnerability to infections by different pathogens such as bacteria and viruses (4, 8). Consequently, IEI patients are considered as a risk group for developing severe COVID-19 (4, 7, 9). There is uncertainty about how a specific IEIs could affect the immune response and outcomes during SARS-CoV-2 infection (4). Such information could be used to optimize the prevention and management approaches for each IEI group. They also reveal key information about the function of the involved genes in host antiviral response and immunopathogenesis of COVID-19 (4).

This Research Topic features two review papers, three original investigations, and a case report. We received 10 submission and accepted 6 papers after a rigorous peer review process. The accepted papers included 100 contributors (Hsu et al., Chen et al., Rovito e al., Milota et al. Scarpa et al, and Clemente et al.).

The role of cytokines and chemokines by innate and adaptive immune responses against SARS-CoV-2 infection was discussed in an extensive review by Hsu et al. Placenta-derived mesenchymal stem cells (pcMSCs) therapy to control the cytokine secretion during severe COVID-19 was proposed by Hsu et al., and investigated by Chen et al. A second review by Rovito et al., explained the contribution of viral and host factors to COVID-19 severity. Risk Factors for development of Severe COVID-19 in patients with IEIs was reported by Milota et al. Another retrospective cohort study by Scarpa et al. explained the impact of hypogammaglobulinemia on the course of COVID-19 in a non-intensive care setting. This was followed by a case report of critical SARS-CoV-2 infection in a pediatric patient with APDS2 Immunodeficiency that was described by Clemente et al.

In a study by Chen et al., the safety and efficacy of pcMSCs treatment were evaluated in a group of five patients with severe COVID-19 who were refractory to standard care and immunosuppressive therapies. The treatment significantly improved the PaO2/FiO2 ratio and Murray’s lung injury scores, and reduced the serum ferritin, lactate dehydrogenase (LDH), and C-reactive protein (CRP) levels. Therapy also suppressed the inflammatory cytokines, increased Treg cells, decreased monocyte and plasma cells, and skewed CD4+ T cells and CD19+ B cells toward adaptive immune responses. Overall, the treatment was effective in preventing mortality by controlling the hyper-inflammatory states of the innate immune response to COVID-19 infection.

Rovito et al. reviewed multiple viral factors and host factors that could contribute to immunopathology and severity in COVID-19. In addition, the authors expanded the factors to include the role of microbiota and impact of lung and gut dysbiosis on immune response to SARS-CoV-2. The viral factors addressed viral inoculum, spike mutations and viral interference of host IFN pathways, while host factors included smoking, sex, age, vitamin D levels, and host genetic factors that comprised the inborn error of immunity which was the focus of current Research Topic. The net effect of viral, host, as well as microbiota factors will then determine the severity levels of COVID-19 that could range from asymptomatic to severe or critical infection.

Milota et al. assessed the risk of severe COVID-19 and hospital admission in a large cohort of patients with IEI. The most common IEI groups with COVID-19 infection were common variable immunodeficiency (CVID), hereditary angioedema (HAE), and unclassified primary antibody deficiency (unPAD). Patient with IEI, except the HAE group, were found to have a 2.3-times increased risk for hospital admission (95%CI: 1.44–3.53) and a higher mortality ratio (2.4% vs. 1.7% in the general population). Lymphopenia and hypogammaglobulinemia were the main predisposing factors to severe SARS-CoV-2 infection among IEI patients. The COVID-IEI patients presented with high seroconversion rate and they responded well to treatments of anti-spike SARS-CoV-2 monoclonal antibodies and convalescent plasma.

Scarpa et al. conducted a retrospective study to evaluate the impact of hypogammaglobulinemia on the course COVID-19 in a non-intensive care setting. Hypogammaglobulinemia was detected in 10.4% of patients admitted to an internal medicine unit with a diagnosis of COVID-19 pneumonia. Around one-fourth of these cases where newly diagnosed identified through a routine serum protein electrophoresis evaluation upon hospitalization. This study included both primary and secondary hypogammaglobulinemia where the antibody deficiencies were due to unknown cause or hematological and solid malignancies, respectively. Hypogammaglobulinemia was associated with higher need for ventilation, higher score of COVID-19 severity, more super infections, and more frequent admission to intensive care unit. This study underlined the add-value of routine serum protein electrophoresis assessment and immunoglobulin replacement therapy (IgRT) in patients admitted with COVID-19.

Clemente et al. described a case report of a 4-year-old boy with an activated PI3K delta syndrome type 2 (APDS2) who developed a critical COVID-19 that resulted in admission to the pediatric intensive care unit (PICU). The patients was a known case of APDS2 with novel heterozygous mutation in PIK3R1 (NM_181523.2:c.243A>T p.(Lys81Asn). Patient had a history of recurrent respiratory infections, lymphadenopathy, and splenomegaly which were characteristic of APDS2 syndrome. He was successfully treated with remdesivir, nitazoxanide, high-dose corticosteroids, and tocilizumab and made a full recovery.

In conclusion, this special issue describes the pathophysiology of COVID-19 encompassing the innate and adaptive immune dysregulation, lymphocytopenia, and cytokine storm (2, 7). The viral and host factors, including the host genetic factors and presence of IEI, could determine the net immune response to SARS-CoV-2 infection elucidating why the disease presentation and severity range from asymptomatic to life-threatening disease (3, 7). The presence of inborn error of immunity could or not increase the risk of infection and severity of COVID-19. Future studies with larger number of patients with different IEIs are needed to determine the impact of an immune defect on susceptibility, severity, and treatment response to SARS-CoV-2 infection.

## Author contributions

RH wrote the first draft. RH, FP, and SA-M provided critical comments and editorial suggestions for revisions. All the authors agreed on the submitted version.

## Funding

Rabih Halwani was supported by the University of Sharjah (COV19-0307) and Al Jalila Foundation (AJF202019).

## Acknowledgments

The guest editors wish to thank all the authors and reviewers for their valuable contributions to this Research Topic.

## Conflict of interest

The authors declare that the research was conducted in the absence of any commercial or financial relationships that could be construed as a potential conflict of interest.

## Publisher’s note

All claims expressed in this article are solely those of the authors and do not necessarily represent those of their affiliated organizations, or those of the publisher, the editors and the reviewers. Any product that may be evaluated in this article, or claim that may be made by its manufacturer, is not guaranteed or endorsed by the publisher.
